# Assessing the integrity of the cognitive processes involved in belief reasoning by means of two nonverbal tasks: Rationale, normative data collection and illustration with brain-damaged patients

**DOI:** 10.1371/journal.pone.0190295

**Published:** 2018-01-30

**Authors:** Aurélie Biervoye, Gaëlle Meert, Ian A. Apperly, Dana Samson

**Affiliations:** 1 Psychological Sciences Research Institute, Université catholique de Louvain, Louvain-la Neuve, Belgium; 2 Institute of Neuroscience, Université catholique de Louvain, Brussels, Belgium; 3 University of Birmingham, Birmingham, United Kingdom; University of Plymouth, UNITED KINGDOM

## Abstract

Every day, we engage in social interactions with other people which require understanding their as well as our own mental states. Such capacity is commonly referred to as Theory of Mind (ToM). Disturbances of ToM are often reported in diverse pathologies which affect brain functioning and lead to problems in social interactions. Identifying ToM deficits is thus crucial to guide the clinicians in the establishment of adequate rehabilitation strategies for patients. Previous studies have demonstrated that ToM is not a unitary function yet currently there are very few standardized tests which allow identifying the type of cognitive processes affected when a patient exhibits a ToM deficit. In the current study, we present two belief reasoning tasks which have been used in previous research to disentangle two types of processes involved in belief reasoning: self-perspective inhibition and the spontaneous inference of another person’s belief. A three-step procedure was developed to provide clinicians with the tools to interpret the patients’ performances on the tasks. First, these tasks were standardized and normative data was collected on a sample of 124 healthy participants aged between 18 and 74. Data collected showed a decrease in performance as a function of age only in the task that loaded most in spontaneous other-perspective demands. There was however no effect of gender or educational level. Cut-off scores to identify deficits were then calculated for the different age groups separately. Secondly, the three-step procedure was applied to 21 brain-damaged patients and showed a large diversity of profiles, including selective deficits of the two targeted ToM processes. The diversity of profiles shows the importance to take into account the multiple facets of ToM during the diagnosis and rehabilitation of patients with suspected ToM deficits.

## Introduction

As social beings, we constantly interact with other people around us. Such interactions require us to reason about mental states such as beliefs, emotions and intentions in order to understand and predict the behavior of others and adapt our own behavior accordingly, an ability usually referred to as Theory of Mind (ToM) [1]. ToM can be impaired following diverse psychiatric and neurological pathologies which affect brain functioning such as autism [2–3], schizophrenia [4], alcohol dependence [5], acquired focal brain lesions such as head injury or stroke (for a review, see [6]), or neurodegenerative diseases (for a review, see [7]). Furthermore, ToM impairments generate major repercussions in the interpersonal relations of the patients [8]. They are thus increasing appeals to improve the clinical assessment to detect ToM deficits (see for example [9]). This is in line with the more general recognition within the fifth edition of the American Psychiatric Association’s Diagnostic and Statistical Manual for Mental Disorders (DSM‑5) [10], of social cognition as one of the six core neurocognitive functions to assess.

The diagnosis of ToM deficits is currently quite challenging for different reasons. Firstly, it is increasingly recognized that ToM is not a unitary function. For example, neuroimaging studies have shown that ToM is sustained by a large brain network including the temporal poles, the temporo-parietal junction, the lateral and median prefrontal cortex as well as the posterior cingulate, with each brain region being likely to play a different contributing functional role during ToM reasoning (for meta-analyses, see [11–12]) In parallel, neuropsychology studies have demonstrated dissociations in patients’ performances across different ToM questions/tasks, providing converging evidence that ToM is composed of different cognitive processes that can be selectively impaired in a patient [13–14]). This highlights the necessity to include in the diagnosis diversified tests that are able to capture the different components relevant for ToM reasoning, in order to better characterize the origin of a patient’s deficit and choose the most appropriate intervention strategy [9]. Table 1 provides examples of existing ToM tests as a function of the ToM components that these tests assess. The components relate to the nature of the mental state inferred (e.g., emotions, desires, intentions, beliefs) and the types of cognitive operation that need to be applied to infer the specific mental state content. The latter components include the distinction between the decoding of expressed mental states and the inference of mental states from situational information. The decoding of others’ mental states relies on immediately available observable cues of a person’s mental state from the person’s facial expressions and eye gaze direction for example while the inference of mental states relies on the inference of the impact of unfolding events for a person’s mental state [15–16]. As we will review later, it has been recently proposed that the inference of mental states can be further decomposed into distinguishable cognitive operations, including the inhibition of one’s own perspective [17–18], the selection of the relevant cues in the environment [19] and the spontaneous tracking of the other person’s perspective [20]. We clarify that the first-perspective relates to the Self, and thus to first person ToM, and that third person perspective refers to Other- and thus to third person ToM.

**Table 1:** Examples of tests used in the diagnosis of ToM deficits.

	Decoding	Inference	
		Non-differentiated	Differentiated
Emotions	Emotion recognition tasks^1^ [23–24] (N: Yes, C: No)		
Desires/Intentions		Attribution of intentions in the Comic-Strip Task [26] (N: No, C: Yes). Task contrasting desire and intention reasoning [27] (N: No, C: Yes).	
Beliefs		Tasks based on the classical paradigm of false beliefs [2], such as the ToM-15^2^[28] (N: Yes, C: Yes)	Tasks reported in the current article and distinguishing self-perspective inhibition and spontaneous other-perspective taking. The Nosy Neighbor Task [19]. distinguishing self-perspective inhibition and the inhibition of distracting information in the environment (N: No, C: Yes)
Mixed mental states	Reading the Mind in The Eyes Test [25] (N: Yes, C: No); Yoni Task [14] (N: No, C: Yes);	Faux-Pas Test [29] (N: Yes, C: Yes) Strange Stories [30–31] (N: Yes, C: Yes)	

Decoding = the person’s mental state can be read out by immediately observable cues (e.g. facial expression); Inference = the person’s mental state is derived from the impact of unfolding events for that person; N = Norms, C = Control items or questions. Control items and questions do not involve the inference of other’s mental states but evaluate other general cognitive functions.

^1^Ekman faces: emotion labelling and emotion discrimination and Facial Expressions of emotions (FEES)

^2^Task and normative data collection in French.

The semi-structured interviews have also been recently designed to assess different facets of ToM (see for example, the Theory of Mind Assessment Scale [21–22]).

Secondly, only few tests have established norms (see Table 1 for examples). Normative data collection on a representative sample of healthy participants is particularly important as there are known inter-individual differences in ToM abilities within the healthy population (e.g., [32–33]) which makes it difficult to establish the threshold for an individual’s task performance to be considered as pathological. There are also reported effects of age (for a meta-analysis, see [34]) or gender ([35–37], but see [38]) within the healthy population which require careful consideration of these demographic factors during the normative data collection.

Finally, ToM tests are usually quite complex because of the very nature of ToM reasoning which requires taking into account multiple and complex sources of information as input to the reasoning. For example, reasoning about someone’s beliefs requires to take into account unfolding events presented visually or verbally about what that person has and has not seen or has being told or not over a certain period of time. Thus, the processing of the input information usually taxes visual and attentional processes, working memory and executive function as well as language processes which can also be affected in the patients. Impaired performance on a ToM task may thus also be linked to diminished cognitive abilities to deal with the processing of the input information. In those cases, the integrity of the core ToM reasoning processes cannot be established but one may still expect difficulties in the patients’ everyday life activities since everyday life ToM reasoning is also based on complex input information. This highlights the importance of including control items which allow assessment of the patients’ abilities to deal with the kind of input information necessary for ToM reasoning (see Table 1 for examples).

One paradigm which has often been used for ToM assessment is the false belief paradigm designed by Wimmer and Perner [39]. In the original version of the test [39], the critical false belief story starts by describing a character’s knowledge about the state of affairs (for example, that a chocolate bar is in the blue cupboard) and then describes a change in the state of the world that the character does not witness (for example, that while the character is away, the chocolate bar is displaced to the green cupboard). The test question then probes participant’s ability to infer that the character’s belief about the state of affairs is now false (e.g., the character wrongly thinks that the chocolate bar is still in the blue cupboard). While the false belief paradigm has often been criticized when used as litmus test to assess the integrity of ToM abilities (i.e. when considered as representative test of all ToM abilities; [40]), its contribution alongside other tests for ToM assessment has stood the test of time as it is particularly sensitive to identify ToM deficits. Indeed, the ability to infer false beliefs is one of the later abilities to develop in children [41] and reasoning about such mental states recruits particularly effortful reasoning processes [42] at least when explicit false belief reasoning is probed (for cases of false belief tasks that appear less effortful, see for example [43–44]). This explains the sensitivity of the classic false belief paradigm even though impaired false belief reasoning does not necessarily mean that all ToM abilities are impaired.

Previous neuropsychological studies reporting dissociations in brain-damaged patients’ performance highlighted the separability of different types of inferential processes which are involved in belief reasoning (and also in reasoning about other mental states). The first type of process is the ability to resist interference from one’s own perspective. In daily life, we often hold a different view to other people (we have different desires, emotions etc.). Thus, correctly reasoning about other people’s mental states requires to putting aside our own point of view. More particularly when we reason about someone else’s belief about the state of affairs, we usually know the real state of affairs. In those circumstances, resisting interference from the knowledge of reality can be challenging, even for healthy adults [45]. Since the first cognitive models of ToM, authors have incorporated an inhibitory processing component that handles the conflict that arises in those circumstances (see for example, the Theory of Mind Mechanism Selection Processor proposed by Leslie and his colleagues [46–47] which is described as an executive control process that inhibits the content of the belief selected by default and changes the focus of attention to the most appropriate belief content). Evidence from neuropsychology has shown that there are particular neural and functional mechanisms that deal with the inhibition of one’s own perspective. This process of inhibition has been illustrated by the case of patient WBA who suffered a brain damage to the right lateral prefrontal cortex following a stroke and who was unable to infer someone else’s mental state (including beliefs and desires) as long as he himself held a strong view (i.e., as long as he knew the real state of affairs or held a conflicting desire; for more details, see [17–19]). By contrast, the patient was able to infer correctly the mental states of others when the demands in self-perspective inhibition were reduced, showing that the deficit was very selective. Interestingly, it has recently been shown that self-perspective inhibition does not only involve domain-general executive processes but may instead recruit, at least in part, executive processes more specific to perspective taking [18] (for a review see [48]). Furthermore, the role of the right lateral prefrontal cortex in self-perspective inhibition has been corroborated with evidence from neuroimaging studies in healthy adults [49–53].

A deficit in self-perspective inhibition is, however, not the sole origin of belief reasoning difficulties in patients. Several patients with lesions to the left temporo-parietal junction were shown to be insensitive to manipulations of self-perspective inhibition demands indicating that the origin of their deficits was unrelated to self-perspective inhibition [19–20; 54–55]. A recent study identified the origin of the deficit in some of these patients as resulting from an inability to spontaneously infer or take into account other people’s beliefs [20]. More specifically, patients KV and IM were unable to take into account in their reasoning that another person has a false belief when the question did not directly ask them to pay attention to the other person’s mental state. In contrast, when the patients were explicitly asked what the other person would do or what the other person thought, the patients were perfectly able to infer the other person’s false belief. Again such dissociation highlights the selectivity of the deficit.

The distinction between self-perspective inhibition and spontaneous other-perspective taking is not only relevant for cases of patients with focal acquired brain-damaged. For example, in the case of patients diagnosed with dementia, it has been shown that patients with the behavioral variant frontotemporal dementia (bvFTD) have a selective impairment in self-perspective inhibition, while this was not the case for patients suffering from Alzheimer’s disease [56]. Selective difficulties in self-perspective inhibition have also been highlighted in older adults [57]. In contrast, deficits in spontaneous other-perspective taking have been documented in autism [58] and in individuals with alcohol dependence [59].

Deficits in self-perspective inhibition will manifest themselves differently in everyday life than deficits in the spontaneous inference of the other perspective. In the former case, we can expect the patients to be extremely egocentric and self-centered which will most likely generate inter-personal conflicts. In the case of a deficit in spontaneous other-perspective inference, we can expect the patients to often miss out on subtle hints about other people’s mental states which may increase the likelihood of misunderstandings during social interactions. Given the different nature underlying each of these types of deficits, we can also expect different interventions to be needed to best fit with the patients’ difficulties.

The diversity of clinical populations for whom the distinction between self-perspective inhibition and spontaneous other-perspective taking is relevant, the important negative effects of deficits to one or the other type of process for successful social interactions, the specific and differential interventions that such deficits may require highlight how essential it becomes to have good clinical tools to assess the integrity of these processes.

The aim of the current study was to optimize two existing belief reasoning tasks in order to make them suitable for clinical assessment. Those two tasks have been previously used for fundamental research purposes in order to distinguish self-perspective inhibition deficits from deficits in spontaneous other-perspective inference. The clinical adaptations consisted in reducing the length of administration, collecting normative data and standardizing the interpretation of the performance. We first describe the sample of participants (healthy participants and brain-damaged patients) and the shortened version of the tasks. We then present and explain the three-step procedure to classify the performance of patients. Finally, we report the results of the normative data collection and the results of the classification on a sample of patients with brain damage. The research protocol was approved by the biomedical ethics committee of the Cliniques universitaires Saint-Luc in Brussels (registration numbers B403201316188 and B403201112043).

## Method

### Participants

#### Healthy participants

One hundred twenty-five healthy participants have been tested for the normative data collection phase of the two belief reasoning tasks. The inclusion criteria were: being aged between 18 and 74 years, to speak French, and not to suffer from a known neurological condition. The participants were recruited by diverse advertisements distributed to the pool of volunteers at the Université catholique de Louvain and via social media. All the participants gave their written consent and received a small financial compensation for their participation. A short neuropsychological examination was performed for participants above the age of 49 in order to exclude potential participants who have impaired cognitive abilities. This neuropsychological examination included the Mini Mental State Evaluation (MMSE) [60], the Ten Word-List Recall from the Consortium to Establish a Registry for Alzheimer's Disease (CERAD) [61], the Trail Making Test [62], the Category Fluency Test and the Letter Fluency Test [63] and the LEXIS Naming Test [64]. Participants above 49 years of age who obtained a score that was 2 standard deviation below the norms on at least two of the neuropsychological tests were excluded from the sample (*n* = 1). The final sample thus included 124 participants (see Table 2 for the participants’ characteristics). Based on a previous study [28], the participants were spread across four age groups (18–34 years, 35–49 years, 50–64 years and 65–74 years). The participants were also divided into two groups according to their socio-educational level: participants who have accomplished 12 years of education or below (level 1) and participants who have accomplished more than 12 years of education (level 2). Moreover, the participants were split according to gender.

**Table 2:** Characteristics of the healthy participants (*n* = 124) for the normative data collection phase of the two belief reasoning tasks.

	Socio-educational level 1		Socio-educational level 2	
	Women	Men	Women	Men
18–34 years (*n* = 34)	8	8	10	8
35–49 years (*n* = 30)	8	7	8	7
50–64 years (*n* = 33)	9	8	8	8
65–74 years (*n* = 27)	8	3	8	8

#### Brain-damaged patients

Twenty-one brain damaged patients (13 women and 7 with a socio-educational level 1, mean age = 57.66 (*SD* = *11*.*80*), age range (min-max) = 38–74 years) participated in this second phase of our study. The brain lesions of the patients were of various etiologies: stroke (*n* = 11), brain tumor (*n* = 2), cerebral anoxia (*n* = 1), encephalitis meningitis (*n* = 1), degenerative dementia of Alzheimer’s type (*n* = 6). This group of patients was heterogeneous but it is representative of the population of brain damaged patients who consult in clinical neuropsychology. The exclusion criteria were the presence of a hemianopia or neglect, as well as major language comprehension deficits. These cognitive deficits were detected by means of standardized tests conventionally used in the neuropsychological diagnostic protocol by the clinicians from the Cliniques universitaires Saint-Luc. The patients were recruited from the Cliniques universitaires Saint-Luc, Brussels. All the patients gave their written consent and received financial compensation for their participation.

### Description of the tasks

Two belief reasoning tasks were used. These non-verbal tasks were created by Apperly and Samson [17, 54–55]. The original tasks included a total of 120 trials which made it unfeasible to use these tasks as clinical tools for time constraint reasons. The number of trials was thus reduced to achieve a total of 48 trials. A subset of selected patients performed the long and original version of the tasks and the interpretation of their profile was compared with the interpretation derived when we only took into account the reduced number of trials. The interpretation was very similar and we thus decided to proceed with the shortened version.

For each task, the instructions were explained to the participants before starting the tasks. Practice trials, which did not include false beliefs, preceded the test trials to ensure that participants understood the instructions. During the test trials, feedback consisting of a photograph depicting the correct answer was presented after each trial to ensure that participants were aware of their incorrect reasoning and were given the opportunity to rectify their reasoning on the next trials. The trials were presented in pseudo-random order (ensuring that there were never more than three trials of the same type one after the other).

#### Reality-unknown belief reasoning task

The first task, labelled as « reality-unknown » or « low inhibition » task in previous studies [54–55], was an adaptation of the non-verbal false belief task designed by Call and Tomasello [65]. It consisted of non-verbal short videos inserted within a PowerPoint presentation (Table 3, see also [20]). For each video, the participant’s task was to find the location of a green object. It was explained that the woman in the video is there to help participants find the location of the green object by using a pink marker that she will put on top of one of two opaque boxe.

**Table 3:** Description of the sequences of events of the different trial categories in the « reality-unknown » belief reasoning task.

Categories of trials	Description of the sequences of events of the reality-unknown videos					
**FB**	S1: The man hides the green object in one of two boxes.	S2: The man shows to the woman where the green object is located.	S3: The woman leaves the room.	S4: During the absence of the woman, the man swaps the boxes.	S5: The woman returns in the room.	S6: The woman indicates one of two boxes with the pink object.
**TB**	S1: The man hides the green object in one of two boxes.	S2: The man shows to the woman where the green object is located.	S3: The woman leaves the room.	S4: **During the absence of the woman, the man lifts the boxes without swapping them.**	S5: The woman returns in the room.	S6: The woman indicates one of two boxes with the pink object.
**MC**	S1: The man hides the green object in one of two boxes.	S2: The man shows to the woman where the green object is located.	S3: **The woman indicates one of the two boxes with the pink object.**	S4: The woman leaves the room.	S5: During the absence of the woman, the man swaps the boxes.	S6: The woman returns in the room.
**Fillers**	S1: The man hides the green object in one of two boxes.	S2: The man shows to the woman where the green object is located.	S3: **The woman indicates one of the two boxes with the pink object.**	S4: The woman leaves the room.	S5: **During the absence of the woman,** **the man moves the object from one of the boxes to the other box (visible transfer)**	S6: The woman returns in the room.

Note that in all the first sequences (S1), the location of the object is not visible to the participants because of the camera’s angle of view. FB = false belief trials, TB = true belief trials, MC = memory control trials, S = Sequence. The changes made in the different categories of trials compared to the false belief category are **in bold**. Compared to FB trials, in the TB trials, the location of the green object is not changed. In the MC trials, the participant knows the object’s location at the beginning of the trial but needs to keep this information in working memory and update it when the man swaps the boxes. In the filler trials, the man makes a visible transfer of the green object which can therefore be seen by participants. This allows participants to realize that the woman genuinely tries to help since participants can directly see that the box the woman pointed at is the true location of the green object. For more details about these trials, see [55].

At the beginning of each video, participants can see a man hiding a green object in one of the two opaque boxes. The man shows to the woman, sitting next to him, where the green object is located. While the woman can see the location of the green object; the angle of the camera is such that participants cannot see in which box the green object is located. In the false belief trials, the woman then leaves the room. During her absence, the man swaps the boxes. The woman comes back in the room, sits down and puts the pink marker on top of one of the boxes. At that point the video is stopped and participants have to point to the box which contains the green object. To answer correctly, participants have to understand that the woman has a false belief about the location of the green object (because the boxes were swapped during her absence), that she therefore put the marker on top of the incorrect box and that therefore the green object is in the other location. Importantly, because participants do not directly witness where the object is placed originally, when they infer that the woman has a false belief, they have no competing knowledge of the real location of the object. It is only after they have inferred the false belief that they can know where the object is. The demands in terms of self-perspective inhibition are therefore reduced during the belief reasoning operations.

The aim of this task is to assess the ability of the participants to infer spontaneously the belief of others, i.e. without explicit instructions to do so. Indeed, the question asked at the end of each video (« show me where the green object is ») directs participants’ attention to the location of the object and not to the mental states of the woman. To check that a deficit in this task is specifically caused by belief reasoning difficulties, different categories of trials other than false belief trials were integrated within the task (see Table 3). The task consists of a total of 24 trials, with 8 false belief trials, 4 true belief trials, 9 memory control trials and 3 filler trials. Memory control and filler trials allow identifying belief reasoning difficulties which would be linked to difficulties with processing the input information (due for example attentional, memory or comprehension problems). True belief trials allow checking that a good performance on the false beliefs trials was not caused by the application of superficial strategies based on the feedback received (such as for example systematically choosing the box opposite to the box the woman pointed at). While such strategies could lead to a correct response on false belief trials, they would however lead to an incorrect response on the true belief trials.

#### Reality-known belief reasoning task

The second task, labelled as « reality-known » or « high inhibition » in previous studies [17], was adapted from classic false belief scenarios such as those originally designed by Wimmer and Perner [39]. This task is relatively similar to the « reality-unknown » belief reasoning task, except that this time, participants have knowledge of the object location throughout the video. The videos also include a male and a female actor (with a different identity to the actors involved in the previous task). At the beginning of each video, the man places the green object in one of the two opaque boxes in full view of both the woman and participants. In the false belief trials, the woman then leaves the room and during her absence, the man opens the boxes to make a visible transfer of the green object from one box to the other. The woman then returns, sits down and looks at a neutral location in the middle of the two boxes. The video then stops and participants are asked to point to the box that the woman will open first to find the green object. To answer correctly, participants have to put aside their own knowledge about the true location of the green object and consider that the woman has not seen the transfer of location and therefore wrongly thinks that the green object is still in the old location. Thus, in contrast to the previous task, the demands in self-perspective inhibition are high. The task consists of a total of 24 trials (see Table 4) amongst which 8 false belief trials, 9 true belief/memory control trials (these trials are qualified as both true belief and memory control because they play both roles) and 7 filler trials (see Table 4). The true belief/memory control trials and filler trials aim to assess the participants’ abilities to deal with the input information (these abilities can be impaired by general cognitive problems such as attentional, memory or comprehension difficulties). The true belief/memory control trials allow checking that correct responses on false belief trials really reflect genuine belief reasoning rather than the use of a superficial strategy.

**Table 4:** Description of the sequences of the different trial categories at the « reality-known » belief reasoning task.

Categories of trials	Description of the sequences of events of the reality-known videos			
**FB**	S1: The man puts the green object in one of two boxes, in sight of the woman.	S2: The woman leaves the room.	S3: During the absence of the woman, the man moves the object from one of the boxes to the other box.	S4: The woman returns in the room.
**TB/MC**	S1: The man puts the green object in one of two boxes, in sight of the woman.	S2: The woman leaves the room.	S3: During the absence of the woman, **the man takes out the green object from the box and puts it back in the same box.**	S4: The woman returns in the room.
**Fillers**	S1: The man hides the green object in one of two boxes, in sight of the woman.	S2: **The woman stays in the room.**	S3: **In presence** of the woman, **the man takes out the green object from the box and puts it back in the same box or puts it in the other box.**	S4: The woman stays always in the room.

Note that in all the first sequences (S1), the participants know the location of the object (the placement of the object in one of the two boxes is visible). FB = false belief trials, TB/MC = true belief/memory control trials, S = Sequence. The changes made in the different categories of trials compared to the false belief category are in **bold**. In the TB/MC trials, the man’s manipulation of the object during the absence of the woman does not result in a change of location. In the Filler trials, the woman stays in the room and witnesses all the object manipulations.

The two belief reasoning tasks were administrated to all participants (healthy participants and brain-damaged participants) always in the following order: the *reality-unknown* task before the *reality-known* task. It is important to keep that particular order to keep the demands in spontaneous other-perspective inference high (the reverse order would mean that participants would be primed by the reality-known task instruction to pay attention to the woman’s mental states). The two tasks were administrated in one session for healthy participants (with a short break between the two tasks) and in two separate sessions in brain-damaged participants (to avoid excessive tiredness). The videos were displayed via a Power-Point presentation on a computer. The answers were manually recorded by the examiner. Both tasks and the instructions are made freely available on Figshare: [https://figshare.com/s/753c27e53a1267bc8042]

### Classification procedure

A three-step classification method was designed to classify the patients according to their performance in the two false belief reasoning tasks and in relation to self-perspective inhibition, spontaneous other-perspective inference and the ability to process input information for belief reasoning. It is important to note that what we propose here is a generic rationale. Clinicians are invited to use in addition their clinical observations to refine their interpretation of the patients’ performance, to measure for example the impact of attentional deficits on the task performance.

#### Step 1: Analysis of the performance in the reality-unknown belief reasoning task

**Fig 1:**
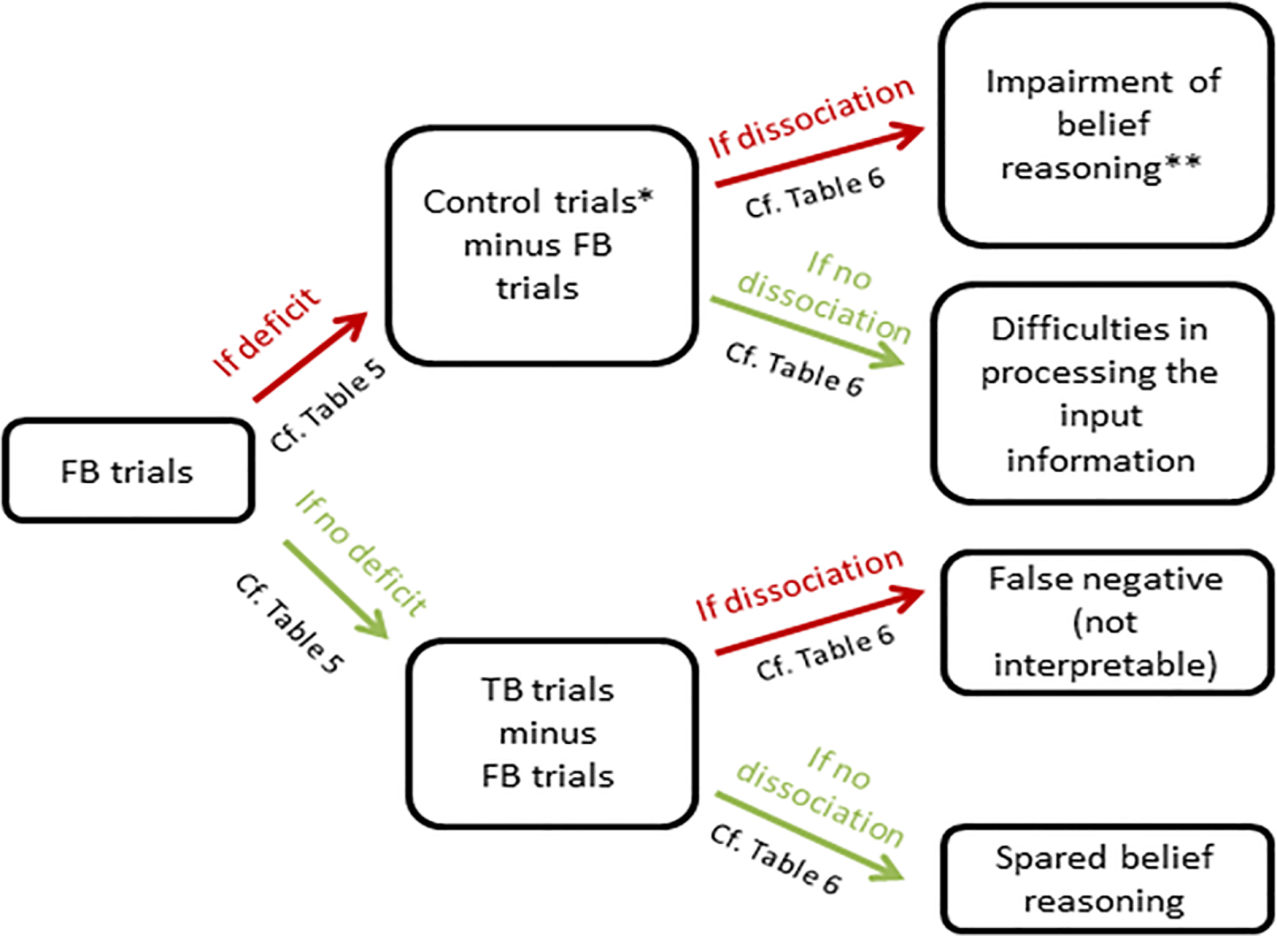
Decision tree to analyze the patient’s profile in the *reality-unknown* belief reasoning task. FB = false belief, TB = true belief, *Control trials include MC (memory control) + Filler trials, **Such deficit could be combined with difficulties in processing the input information if the performance on the control trials is below the cut-off albeit better than the performance on the FB trials.

Three scores were calculated based on the performance on the reality-unknown belief reasoning task: the total number of correct responses on the false belief trials (FB, score out of 8), the total number of correct responses on the true belief trials (TB, score out of 4) and the total number of correct responses on the control trials. This latter score takes into account both the memory control trials (MC) and the filler trials (MC + Fillers, score out of 12). The decision tree schematized in Fig 1 was then used to compare the participant’s scores with the cut-off scores derived from the matched healthy controls’ performance collected in the current study and classify the global profile of the participant in the task.

It is first determined whether the participant has a score for the FB trials that is at or below the cut-off score (mean score of control participants minus 2 standard deviations, cf. Table 5). If the score is below cut-off then the difference between their performance on the FB trials and relevant control trials (i.e., memory control and filler trials) is to be examined to determine whether there is a dissociation between the two scores. The rationale behind this is that if a patient has equivalent difficulty with FB trials and control trials, it means that he has a probable impairment processing the input information, which will naturally lead to errors on FB trials. On the other hand, if a patient makes significantly more errors on the FB trials than on the control trials, this means that he or she probably has difficulties with belief processing per se above any deficit in processing the input information. To establish the possible presence of a dissociation, and because the maximum score was different for FB compared to control trials, the percentage of correct responses (rather than the absolute number of correct responses) for the FB trials has to be subtracted from the percentage of correct responses for the control trials. If this difference equals or exceeds the cut-off value (mean difference in control participants + 2 standard deviations, cf. Table 6), this indicates the presence of a dissociation, indicating likely impairment in belief processing per se. Note however that even if the difference indicates a dissociation, a probable impairment in belief processing could also be combined with difficulties in processing the input information in some cases. For example, for a patient (35 years) who has a performance of 50% for the control trials and 12,5% for false belief trials, we will conclude that the difference between his performance on false belief trials and on control trials is below the cut-off suggesting a difficulties in reasoning about false belief per se. However, his performance on the control trials suggest that he has, in addition, general cognitive difficulties which probably also interfere in belief reasoning. Whether the performance on control trials is below the cut-off score can be checked in Table 5. The absence of dissociation (difference below the cut-off value) demonstrates that the performance on FB trials is probably contaminated with difficulties in processing the input information to belief reasoning (possibly due to other cognitive difficulties such as attentional, memory or comprehension difficulties).

**Table 5:** Cut-off scores (threshold values derived from the healthy controls’ performance collected in the current study; if the participant’s performance is at or below this cut-off score, her/his performance is pathological) for the false belief, true belief and control trials in the *reality-unknown* task and the *reality-known* task.

	Mean	Standard deviation (SD)	Mean minus 2 SD	Cut-off
**FB « *Reality*-unknown» (score out of 8)**				
18–34 years	7.32	*1*.*12*	*5*.*08*	**5**
35–49 years	7.50	*0*.*90*	*5*.*25*	**5**
50–64 years	6.85	*1*.*46*	*3*.*93*	**4**
65–74 years	6.48	*1*.*50*	*3*.*48*	**3**
**Control trials « *Reality*-unknown» (score out of 12)**				
18–34 years	12	*0*.*00*	*12*.*00*	10*
35–49 years	11.9	*0*.*30*	*11*.*30*	10*
50–64 years	11.79	*0*.*48*	*10*.*83*	10*
65–74 years	11.88	*0*.*32*	*11*.*24*	10*
**TB « *Reality*-unknown» (score out of 4)**				
18–34 years	3.94	*0*.*24*	*3*.*46*	2*
35–49 years	3.90	*0*.*40*	*3*.*10*	2*
50–64 years	3.79	*0*.*54*	*2*.*71*	2*
65–74 years	3.63	*0*.*63*	*2*.*37*	2*
**FB « *Reality-known* » (score out of 8)**				
18–34 years	7.94	0.24	7.46	6*
35–49 years	7.87	*0*.*43*	7.01	6*
50–64 years	7.88	*0*.*55*	6.78	**6**
65–74 years	7.85	*0*.*36*	7.13	6*
**Control trials « *Reality-known* » (score out of 16)**				
18–34 years	15.94	*0*.*24*	*15*.*46*	14*
35–49 years	15.96	*0*.*18*	*15*.*60*	14*
50–64 years	15.93	*0*.*35*	*15*.*23*	14*
65–74 years	15.77	*0*.*51*	*14*.*75*	14*
**TB/MC « *Reality-known* » (score out of 9)**				
18–34 years	8.97	*0*.*17*	*8*.*63*	7*
35–49 years	9	*0*	*9*.*00*	7*
50–64 years	8.94	*0*.*35*	*8*.*24*	7*
65–74 years	8.93	*0*.*27*	*8*.*39*	7*

TB = True belief trials. FB = False belief trials. TB/MC = True belief/memory control trials.

* = acceptance of one distraction error. The control trials include memory control and fillers for reality-unknown task. Control trials include true belief/memory control and filler trials for reality-known task. For the false belief and control trials of each task (each expressed on a score out of 8, 12 or 16), the cut-off scores were calculated according to a sequence of criteria: 1) the mean minus 2 standard deviation obtained was rounded mathematically without decimals (e.g., 3,93/8 became 4/8); 2) if this cut-off value does not allow for one distraction error, the value was lowered by one unit to allow for a distraction error (e.g., for a z-score of 7,46/8, the cut-off became 6/8).

**Table 6:** The cut-off scores (derived from the healthy controls’ performance collected in the current study) to determine the presence or absence of a dissociation between two categories of trials.

Cut-offs				
	For the *reality-unknown* task		For the *reality-known* task	
	Control trials minus FB trials	TB trials minus FB trials	Control trials minus FB trials	TB and MC trials minus FB trials
18–34 years	36.5%	-25.1%	7.2%	-11.1%*
35–49 years	27.5%	-19.1%	11.7%	-11.1%*
50–64 years	50.4%	-26.9%	10.7%	-11.1%*
65–74 years	56.2%	-33.2%	11.4%	-11.1%*
Deficit if value is	**=** or **>** than cut-off	**=** or **<** than cut-off	**=** or **>** than cut-off	**<** than cut-off

FB = false beliefs, MC = memory control, TB = true beliefs, control trials include memory control and fillers for reality-unknown task. Control trials include true belief/memory control and filler trials for reality-known task.

* = acceptance of one distraction error on true belief/memory control trials.

The raw scores for each type of trial were transformed in percentages to be on the same scale. To identify potential dissociations between the FB trials and the TB or control trials, the differences between these categories of trials (expressed in terms of percentage of correct responses) were calculated for each control participant. The cut-off score is equivalent to 2 standard deviations away from the mean performance of the control participants by age group. However, if the cut-off value did not allow for a distraction error, the value was lowered to allow for a distraction error.

If the participant has a score for the FB trials that is above the cut-off score (cf. Table 5), than the difference between the performance on the TB trials and the FB trials is to be examined to see whether there is a dissociation between the two scores. The rationale behind this is that if a patient makes significantly more errors on the TB trials than on the FB trials this means that he or she probably uses a superficial strategy to succeed on the FB trials based on the feedback received (such as for example systematically choosing the other box to the box that the woman pointed at). On the other hand, if the patient’s performance is as good on the TB as on the FB trials this means that he or she does not use a compensatory strategy and thus has no difficulties with belief processing. Here again, to establish the possible presence of a dissociation, and because the maximum score was different for FB compared to TB trials, the percentage of correct responses (rather than the absolute number of correct responses) for the FB trials has to be subtracted from the percentage of correct responses for the TB trials. If this difference is equal or below to the cut-off value (mean difference in control participants—2 standard deviations, cf. Table 6), this indicates the presence of a dissociation which is in favor of a profile qualified as possible « false negative » i.e. the subject may have used a superficial strategy to compensate probable difficulties with belief processing.

#### Step 2: Analysis of the performance in the reality-known belief reasoning task

**Fig 2:**
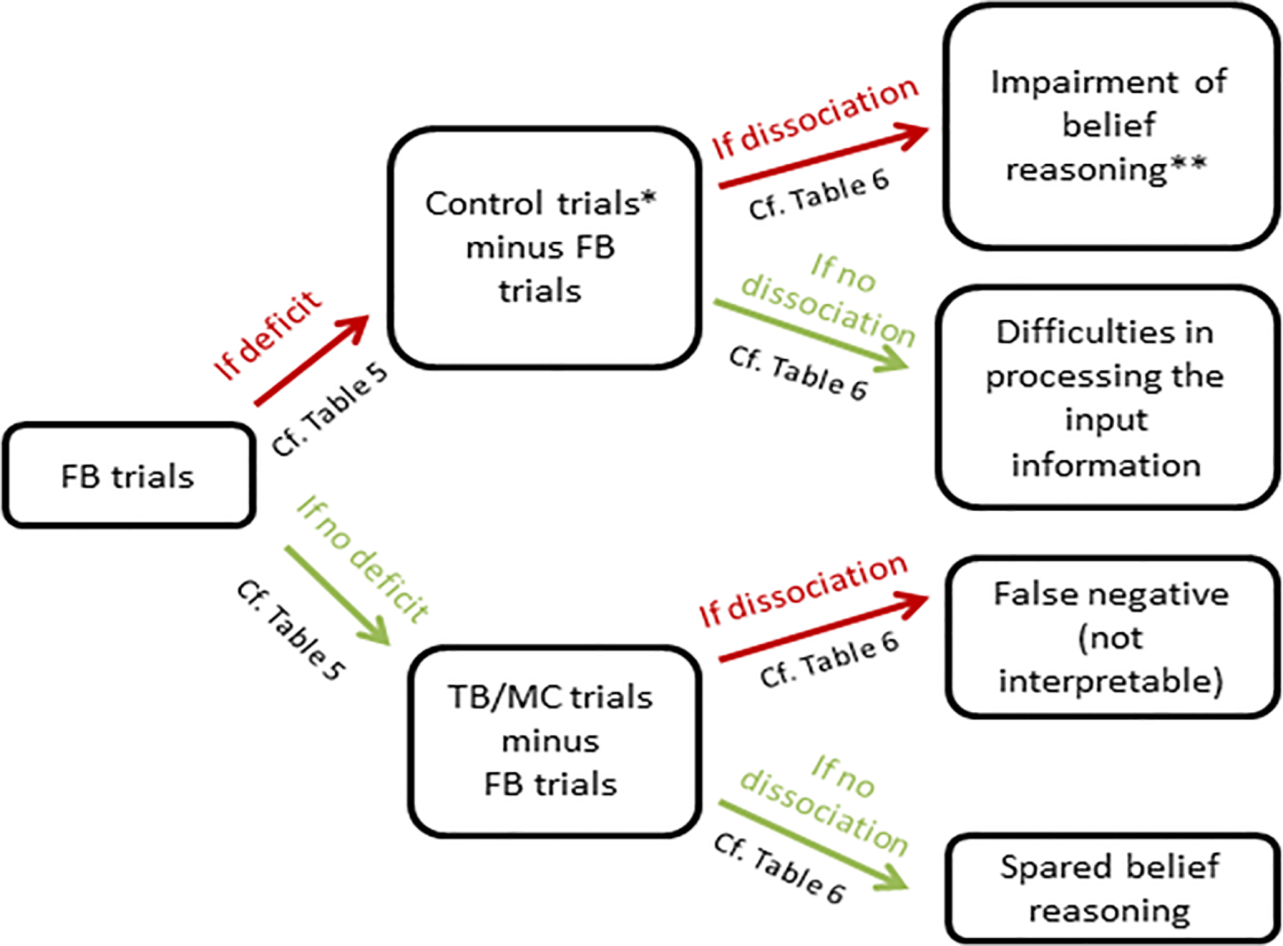
Decision tree to analyze the patient’s profile in the *reality-known* belief reasoning task. FB = false belief, TB/MC = true belief/memory control, *Control trials include TB/MC + filler trials, ** Such deficit could be combined with difficulties in processing the input information if the performance on the control trials is below the cut-off albeit better than the performance on the FB trials.

Three scores were calculated based on the performance on the reality-known belief reasoning task: the total number of correct responses on the false belief trials (FB, score out of 8), the total number of correct responses on the true belief/memory control trials (TB/MC, score out of 9; as a reminder, these trials act as both true belief and memory control trials) and the total number of correct responses on the control trials (TB/MC + Fillers, score out of 16). This latter score takes into account both the true belief/memory control trials (TB/MC) and the filler trials. The decision tree schematized in Fig 2 was then used to compare the participant’s scores with the cut-off scores derived from the matched healthy controls’ performance collected in the current study. This allowed to classify the global profile of the participant in the task.

If the participant has a score for the FB trials that is at or below the cut-off score (cf. Table 5), than the difference between the performance on the control trials (TB/MC + Fillers) and the FB trials is to be examined to determine whether there is a dissociation between the two scores. The rationale behind this is the same as the one described for the reality-unknown version of the task. To establish the possible presence of a dissociation, the percentage of correct responses (rather than the absolute number of correct responses) for the FB trials has to be subtracted from the percentage of correct responses for the control trials. If this difference is equal or above the cut-off value (mean difference in control participants + 2 standard deviations, cf. Table 6), this indicates the presence of a dissociation which is in favor of a probable impairment in belief processing per se. Note also that as for the reality-unknown version of the task, even if the difference indicates a dissociation, a probable impairment in belief processing could be also combined with difficulties in processing the input information in some cases (see Table 5 for the cut-off scores for the control trials). Conversely, the absence of dissociation (difference below the cut-off value) indicates that the performance on FB trials is probably contaminated by difficulties in processing the input information to belief reasoning.

If the participant has a score for the FB trials that is above to the cut-off score (cf. Table 5), than the difference between the performance on the TB/MC trials and the FB trials is to be examined to determine whether there is a dissociation between the two scores. The rationale is again the same as the one described for the reality-unknown version of the task. To establish the possible presence of a dissociation, the percentage of correct responses for the FB trials has to be subtracted from the percentage of correct responses for the TB/MC trials. If this difference is equal to or below the cut-off value (mean difference in control participants—2 standard deviations, cf. Table 6), this indicates the presence of a dissociation which is in favor of a profile qualified as « false negative » i.e. the subject uses alternative strategy to compensate probable difficulties with belief processing. The absence of dissociation (difference above to the cut-off value) demonstrates that the subject has no deficit in belief processing.

#### Step 3: Analysis of the integrity of the ToM processes

Finally, the relative integrity of the participants’ ability to spontaneously infer someone else’s belief and to inhibit their own perspective was examined by comparing the profile obtained for each belief reasoning task (cf. Table 7). The general rationale is the following. Given that each task loads differentially on the spontaneous other-belief tracking and self-perspective inhibition demands, the strongest conclusion of a deficit in spontaneous other-belief tracking can be drawn when the participant exhibits a probable impairment in belief reasoning in the reality-unknown task and shows a classical or strong dissociation between his/her belief reasoning abilities in the reality-unknown versus the reality-known tasks, with more difficulties in the reality-unknown task (cf. Table 8 for the cut-offs for differential scores between the false belief trials in the two tasks). Indeed, in a single-case studies, a classical dissociation is established when the following three criteria are met: 1) the patient has a deficit on Task X, 2) the patient is within normal limits on Task Y and 3) there is a significant difference between performances on Task X and Y. A strong dissociation (or differential deficit) is a weaker version of dissociation and is established when 1) the patient has a deficit on Task X, 2) the patient has also a deficit on Task Y, and 3) there is a significant difference between performances on Task X and Y [66]. Likewise, the strongest conclusion of a deficit in self-perspective inhibition can be drawn when the participant shows a probable impairment in belief reasoning abilities in the reality-known task and the reverse classical or strong dissociation between his belief reasoning abilities across the two tasks.

**Table 7:** Decision table to analyze the integrity of the targeted ToM processes. The interpretations are based on the various possible profiles of performance across the two belief reasoning tasks. According to the patient’s performance profile in the reality-unknown belief reasoning task, the clinician is invited to select the profile of performance in the reality-known task, followed by the type of dissociation across the false belief trials of the two tasks.

**Spared belief reasoning in the RUK task**		
**Interpretation in RK task**	**Dissociation across FB in RUK vs. RK** **(See cut-offs for differential scores in Table 8)**	**Interpretation of integrity of the two target ToM processes**
Spared belief reasoning	n/a	• Spontaneous other-belief processing: spared • Self-perspective inhibition: spared
Impairment of belief reasoning	Yes (= classical dissociation)	• Spontaneous other-belief processing: spared • Self-perspective inhibition: impaired
	No (= no reliable dissociation)	• Spontaneous other-belief processing: spared • Self-perspective inhibition: mildly impaired
Difficulties in processing the input information	n/a	• Spontaneous other-belief processing: spared • Self-perspective inhibition: *Needs re-testing in the RK task when the ability to process the input information has been improved*
False negative	n/a	• Spontaneous other-belief processing: spared • Self-perspective inhibition: *Needs re-testing in the RK task with strategies being explicitly discouraged*
**Impairment of belief reasoning in the RUK task**		
**Interpretation in RK task**	**Dissociation across FB in RUK vs. RK** **(See cut-offs for differential scores in Table 8)**	**Interpretation of integrity of the two target ToM processes**
Spared belief reasoning	Yes (= classical dissociation)	• Spontaneous other-belief processing: impaired • Self-perspective inhibition: spared
	No (= no reliable dissociation)	• Spontaneous other-belief processing: mildly impaired • Self-perspective inhibition: spared
Impairment of belief reasoning	Yes (= strong dissociation)	• Spontaneous other-belief processing: impaired • Self-perspective inhibition: impaired • One process is more impaired than the other (depending on the direction of the dissociation)
	No (= no reliable dissociation)	• *Needs further investigation to see whether both processes are impaired or another ToM process common to both tasks is impaired (such as a loss of knowledge about beliefs)*
Difficulties in processing the input information	n/a	• *Needs re-testing in the RK task when the ability to process the input information has been improved to see whether both ToM processes are impaired or another ToM process common to both tasks is impaired (such as a loss of knowledge about beliefs) or solely the spontaneous other-belief processing is impaired*
False negative	n/a	• *Needs re-testing in the RK task with strategies being explicitly discouraged to see whether both ToM processes are impaired or another ToM process common to both tasks is impaired (such as a loss of knowledge about beliefs) or solely the spontaneous other-belief processing is impaired*
**Difficulties in processing the input in the RUK task**		
**Interpretation in RK task**	**Dissociation across FB in RUK vs. RK** **(See cut-offs for differential scores in Table 8)**	**Interpretation of integrity of the two target ToM processes**
Spared belief reasoning	n/a	• Spontaneous other-belief processing: *Needs re-testing in the RUK task when the ability to process the input information has been improved* • Self-perspective inhibition: spared
Impairment of belief reasoning	n/a	• *Needs re-testing in the RUK task when the ability to process the input information has been improved to see whether both ToM processes are impaired or another ToM process common to both tasks is impaired (such as a loss of knowledge about beliefs) or solely the spontaneous self-perspective inhibition processing is impaired*
Difficulties in processing the input information	n/a	• *Needs re-testing in the RK and RUK task when the ability to process the input information has been improved*
False negative	n/a	• *Needs re-testing in the RUK task when the ability to process the input information has been improved* • *Needs re-testing in the RK task with strategies being explicitly discouraged*
**False negative in the RUK task**		
**Interpretation in RK task**	**Dissociation across FB in RUK vs. RK** **(See cut-offs for differential scores in Table 8)**	**Interpretation of integrity of the two target ToM processes**
Spared belief reasoning	n/a	• Spontaneous other-belief processing: *Needs re-testing in the RUK task with strategies being explicitly discouraged* • Self-perspective inhibition: spared
Impairment of belief reasoning	n/a	• *Needs re-testing in the RK task with strategies being explicitly discouraged to see whether both ToM processes are impaired or another ToM process common to both tasks is impaired (such as a loss of knowledge about beliefs) or solely the slef-perspective inhibition processing is impaired*
Difficulties in processing the input information	n/a	• Spontaneous other-belief processing: *Needs re-testing in the RUK task with strategies being explicitly discouraged* • Self-perspective inhibition: *Needs re-testing in the RK task when the ability to process the input information has been improved*
False negative	n/a	• Spontaneous other-belief processing: *Needs re-testing in the RUK task with strategies being explicitly discouraged* • Self-perspective inhibition: *Needs re-testing in the RK task with strategies being explicitly discouraged*

RK = *reality-known* **belief reasoning task,** RUK = the *reality-unknown* belief reasoning task, FB = false belief items, n/a = not-applicable.

**Table 8:** The cut-off scores (threshold values derived from the healthy controls’ performance collected in the current study; if the difference of participant’s scores is at or above this cut-off score, the difference is pathological), for the difference between the false belief trials of the *reality-known* task and the false belief trials of the *reality-unknown* task.

	Cut-off
	FB trials of the reality-known task minus FB trials of the reality- unknown task
18–34 years	**3**
35–49 years	**3**
50–64 years	**4**
65–74 years	**5**

FB = false belief trials. To determine the presence or the absence of a dissociation between false belief trials of each task, the differences between the FB trials of each task were calculated for each control participant. The cut-off scores were then calculated for each age group following these criteria: the value obtained was rounded at the higher unit (e.g., 4,36/8 became 5/8) to minimise the potential presence of false positives. Because the number of trials was identical across the two tasks, we used here the raw value rather than percentages

When a participant shows a profile conforming to a probable belief reasoning impairment in both tasks and there is no dissociation, then one can only conclude that either both the spontaneous other-belief tracking and the self-perspective inhibition processes are impaired or that the impairment affects a ToM process common to both tasks. For example, one may make the hypothesis that the participant has lost knowledge about the nature of beliefs or about how they are formed. Additional investigation could probe the participant’s ToM knowledge to disentangle these possible interpretations (for example, the clinician may ask questions probing the patient’s knowledge about the nature of beliefs).

When there is a probable belief reasoning impairment in one task but there is either no dissociation with the belief reasoning performance in the other task or the performance in the other task conforms to an input processing impairment or a false negative, then it can only be concluded that the ToM processes assessed in the task in which the patient shows a belief reasoning impairment is “possibly” impaired. Here also additional investigation would be needed to disentangle the possible interpretations.

Finally, whenever belief reasoning is spared in a task, the processes assessed in the task can be interpreted as spared. Table 7 details the interpretations based on the various possible profiles of performance across the two belief reasoning tasks.

## Results

### Healthy participants

We first tested whether performance varied significantly according to the age, the gender and the socio-educational level of the healthy participants in order to specify according to which variable the norms should be computed. For each task, an ANOVA was run on the percentage of correct responses with the type of trials (3 levels for the reality-unknown belief reasoning task: FB trials, TB trials and control trials which include MC and filler trials; 3 levels for the reality-known belief reasoning task: FB trials, TB/MC trials and Filler trials) as a repeated factor, and the age group (4 levels: 18–34, 35–49, 50–64, 65–74), the gender and the socio-educational level (2 levels: below or above 12 years) as between-participant factors. When the sphericity assumption was violated, degrees of freedom were corrected by using the Greenhouse-Geisser correction when the sphericity estimate was lower than .75 and the Huynh-Feldt correction when this estimate was greater than .75 (Girden, 1992, cited by Field, 2005, p. 431 [67]). In order to explain any significant interaction, we ran post-hoc analyses. Finally, in order to test whether the tasks differed in terms of complexity, we ran an ANOVA on the percentage of correct responses on the false belief trials with the task (reality-unknown vs. known) as a repeated factor and the age, the socio-educational level and the gender as between-participant factors.

**Fig 3:**
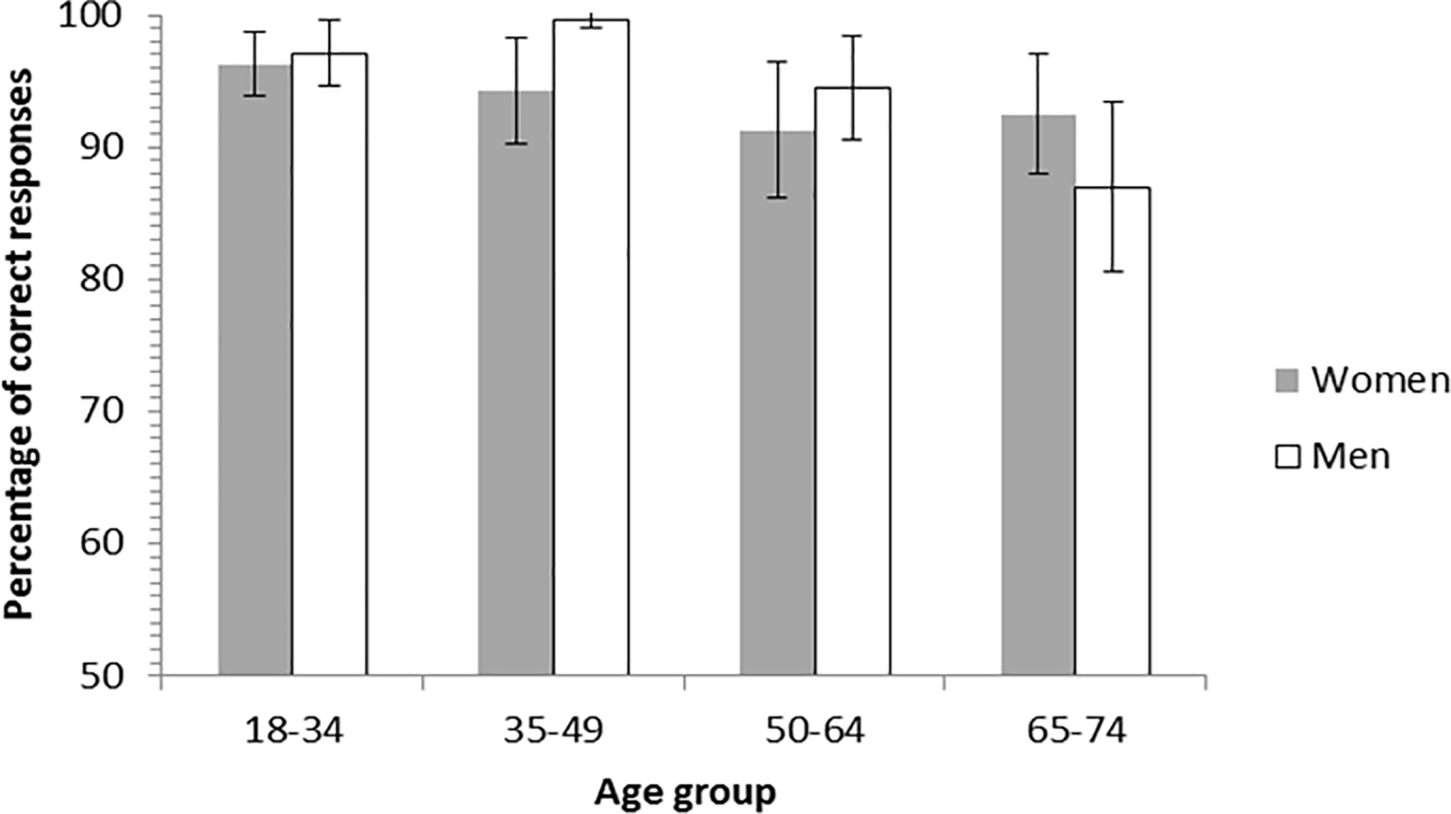
Percentage of correct responses according to age group and gender for the reality-unknown belief reasoning task. Accuracy did not differ significantly between women and men, except in the 35–49 year-olds. The error bars represent the confidence intervals 95% around the mean.

For the reality-unknown belief reasoning task, the main effect of age was significant, *F*(3, 108) = 5.91, *p* < .01, η_p_^2^ = .14. In contrast, neither the main effect of gender nor the main effect of socio-educational level were significant, both *Fs*(1, 108) < 1, *ps* > .10. All the double interactions involving these variables were not significant either (all *p*s >. 10), except the Age by Gender interaction that was marginally significant, *F*(3, 108) = 2.62, *p* = .06. This interaction can be first explained by the fact that performance did not differ between women and men (18–34 y.o.: *t*(32) = —.50, *p* > .10; 50–64 y.o.: *t*(31) = -1.07, *p* > .10; 65–74 y.o.: *t*(25) = 1.59, *p* > .10), except in the 35–49 year olds, *t*(15.75) = -2.85, *p* = .01 (see Fig 3). In this age group, women (*M* = 94.3%, *SD* = 7.5%) performed less accurately than men (*M* = 99.7%, *SD* = 1.1%). The interaction can also be explained by the fact that the effect of Age was not significant in women, *F*(3, 63) = 1.32, *p* > .10, while it was significant in men, *F*(3, 53) = 9.42, *p* < .01, η_p_^2^ = .35. Among men, the 65–74 year-olds (*M* = 87.0%, *SD* = 9.5%) performed significantly less accurately compared to the 18–34 year-olds (*M* = 97.1%, *SD* = 4.7%), *p* < .01, to the 35–49 year-olds (*M* = 99.7%, *SD* = 1.1%), *p* < .01, and to the 50–64 year-olds, (*M* = 94.5%, *SD* = 7.3%), *p* < .01. The 50–64 year-olds also performed significantly less accurately than the 35–49 year-olds, *p* < .05.

**Fig 4:**
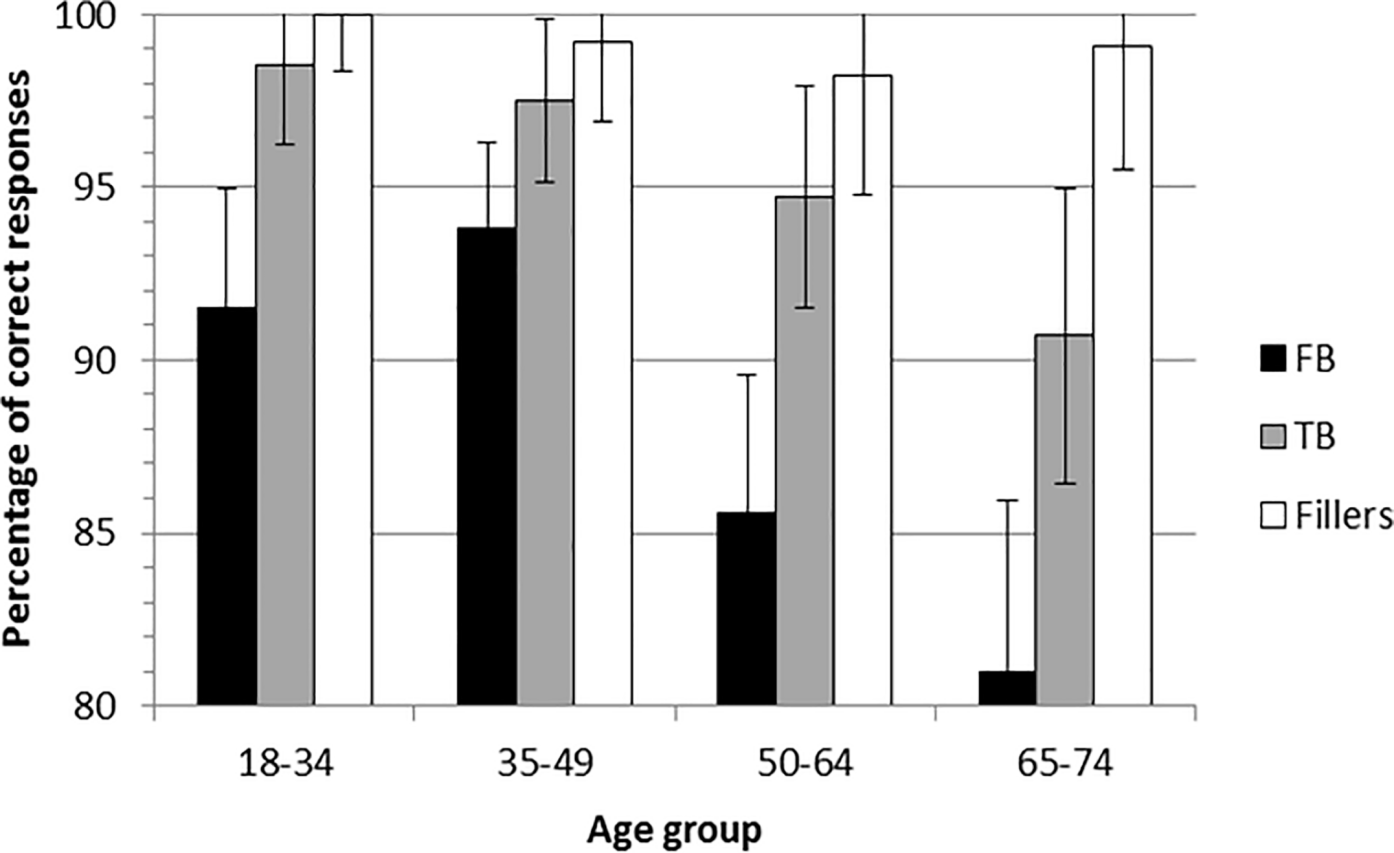
Percentage of correct responses as a function of age group and type of trials for the reality-unknown belief reasoning task. The error bars represent the confidence intervals 95% around the mean, they were corrected for repeated measures by age group according the Cousineau method [68]. FB = false belief trials, TB = true belief trials, Control = control trials (including memory control and Filler trials).

Furthermore, the main effect of the type of trials was significant, *F*(2, 216) = 32.87, *p* < .01, η_p_^2^ = .23. This effect did not interact with gender, socio-educational level or any combination of these variables (all *F*_s_ < 1, *p*_s_ > .10). In contrast, the main effect of the type of trials significantly interacted with the age group, *F*(6, 216) = 2.23, *p* = .04, η_p_^2^ = .06. In order to explain this interaction, we first tested the effect of the type of trials for each age group separately. This effect was significant for all age groups (all *p*s < .05): accuracy was significantly lower for the FB trials than for the TB trials and the control trials (which included MC and filler trials) in all age groups (all *p*s < .05), except in the 35–49 group for which the difference between the FB trials and the TB trials was only marginally significant (*p* = .08). In addition, accuracy was lower for the TB trials than for the control trials (i.e., MC and filler trials) in the 65–74 year-olds, *p* = .01. Second, we tested the effect of the age group for each type of trials separately (see Fig 4). The effect of the age group was significant for the FB trials, *F*(3, 120) = 3.89, *p* = .01, η_p_^2^ = .09. Accuracy was lower for the 65–74 year-olds compared to the 35–49 year-olds, *p* < .01, and to the 18–34 year-olds, *p* = .01, and lower for the 50–64 year-olds than for the 35–49 year-olds, *p* = .04. For the TB trials, the effect of the age group tended to be significant, *F*(3, 120) = 2.58, *p* = .06, η_p_^2^ = .06, with the accuracy being lower in the 65–74 year-olds compared to the 35–49 year-olds, *p* = .03, and to the 18–34 year-olds, *p* = .01. For the control trials (i.e., MC and filler trials), the effect of the age group also tended to be significant, *F*(3, 120) = 2.35, *p* = .08, η_p_^2^ = .06. Accuracy was lower in the 50–64 year-olds compared to the 18–34 year-olds, *p* < .01.

In sum, for the reality-unknown belief reasoning task, global performance did not differ according to the socio-educational level. The global performance did not differ according to the gender of participants, except for one age group. Accuracy significantly decreased with age, especially in men and for the FB trials. Finally, accuracy was lower for FB trials compared to all the others trials as shown in Fig 4.

For the reality-known belief reasoning task, neither the main effect of gender, *F*(1, 108) < 1, *p* > .10, the main effect of socio-educational level, *F*(1, 108) < 1, *p* > .10, nor the main effect of age group, *F*(3, 108) < 1, *p* > .10, were significant. All the double and triple interactions involving the gender, the age group and/or the socio-educational level did not reach the statistical level of significance (all *p*s >. 10). The main effect of the type of trials (FB vs. TB/MC vs. Filler) was not significant either, *F*(1.60, 172.27) = 2.342, *p* = .111, and did not interact with the gender, the socio-educational level, the age group or any combination of these variables (all *p*_s_ > .10). In sum, performance in the reality-known belief reasoning task did not vary significantly according the age, the gender, the socio-educational level or the type of trials.

Finally, we tested the relative complexity of the two belief reasoning tasks by running an ANOVA on the percentage of correct responses on the FB trials with the task (reality-unknown vs. known) as a repeated factor and the age group, the gender and the socio-educational level as between-participant factors. Regarding the between-participant factors, the main effect of age was significant, *F*(3, 108) = 3.57, *p* = .02, η_p_^2^ = .09, while the main effect of gender and the main effect of socio-educational level were not significant, both *Fs*(1, 108) < 1, *p* > .10. The two-way and the three-way interactions were not significant [age * gender: *F*(3, 108) = 1.11, *p* >. 10; age * socio-educational level: *F*(3, 108) < 1, *p* > .10; gender * socio-educational level: *F*(3, 108) < 1, *p* > .10; age * gender * socio-educational level: *F*(3, 108) < 1, *p* > .10] Regarding the repeated factor and its interaction with the between-participant factors, the main effect of the task was significant, *F*(1, 108) = 59.35, *p* < .01, η_p_^2^ = .35, with accuracy being lower in the reality-unknown FB trials (*M* = 88.2%, *SD* = 16.4%) than in the reality-known FB trials (*M* = 98.6%, *SD* = 5.1%). This effect did not interact neither with gender nor with socio-educational level, both *Fs*(1, 108) < 1, *p* > .10, while it significantly interacted with age, *F*(3, 108) = 4.75, *p* < .01, η_p_^2^ = .12. This two-way interaction was qualified by a significant three way interaction between the task, the age group and the gender, *F*(3, 108) = 2.78, *p* = .045, η_p_^2^ = .07. This interaction was explained by the fact that the effect of the task was significant in each age group in women and men (all *p*s < .05), except in 35–49 year-old men for which there was no significant difference between the two tasks (floor effect: 99.11 vs 98.2%), *t*(13) = 1.00, *p* > .10. All other three-way interactions and the four-way interaction were not significant, all *F*s < 1, *p* >. 10. In sum, these results showed a greater complexity of the reality-unknown FB trials compared to the reality-known FB trials that was showed in all age groups of men and women, except in 35–49 year-old men due to a floor effect.

Altogether, these results showed an effect of age on performance on the reality-unknown especially on FB trials that were also less accurately performed compared to the true belief trials and the control trials, a very limited effect of the gender with significant differences being limited to a specific age group, and a greater complexity of the reality-unknown FB trials compared to the reality-known FB trials that was showed in all age groups of men and women, with only one exception.

Based on these results, only the distribution of the participants according to age group was maintained for the calculation of the norms for the two false belief reasoning tasks. For each trial type, the mean and the standard deviation of the control participants by age group was calculated. The cut-off (threshold values to establish that a score is pathological and are based on the score that is 2 standard deviation away from the mean performance of the control participants) are presented in Tables 5–7. The detailed procedure to calculate the cut-off scores for each type of trial is explained below each table.

### Brain-damaged patients

For each patient, the scores for the different categories of trials were calculated and compared with the norms of age-matched control participants according the classification method described above. The results showed overall a diversity in the profiles of the patients’ performance in the false belief reasoning tasks (cf. Table 9). Overall, 3 out of the 21 brain-damaged patients showed impaired self-perspective inhibition processes while 14 had spared self-perspective inhibition processes. Furthermore, 2 out of the 21 brain-damaged patients showed impaired spontaneous other-perspective-taking while 12 showed spared abilities to spontaneously take into account the other person’s perspective. Then, 3 out of the 21 brain-damaged patients showed equivalent levels of belief reasoning impairment in both tasks which may indicate either that both processes were impaired or that another process common to both tasks was impaired (such as the knowledge about nature of beliefs). However, further clinical examination consisted to administrate to these three patients another ToM task with lower spontaneous other perspective taking demands and lower self-perspective inhibition demands. The performance observed on that additional task allowed us to exclude semantic difficulties for two out of the three patients (since these two patients showed a preserved performance on the belief inference trials of the task). However, the remaining patient was also impaired in this additional task specifically for beliefs; in that latter case, one can suspect that the patient had probably impaired semantic knowledge about beliefs. Finally, for a few patients, the integrity of these two core ToM processes could not be established either because the patients had difficulties processing the input information, because their profile of response suggested the use of a superficial strategy.

**Table 9:** Demographics data, performances and classification of the brain-damaged patients (*n* = 21) at the two false belief reasoning tasks.

			Reality-unknown task				Reality-known task				Global FB profile	
ID	Age/ Sex	Etiology	%corr FB	%corr TB	%corr control trials	Integrity of belief reasoning	%corr FB	%corr TB/MC	%corrcontrol trials	Integrity of belief reasoning	Dissociation in FB performance	Integrity of FB processes
1	46/F	stroke	75	75	75	Spared	87.5	100	100	Spared	n/a	Spont. Other: spared Self Inhibit.: spared
2	67/M	stroke	100	75	100	Spared	100	100	100	Spared	n/a	Spont. Other: spared Self Inhibit.: spared
3	51/F	brain tumor	100	100	100	Spared	100	100	100	Spared	n/a	Spont. Other: spared Self Inhibit.: spared
4	69/M	stroke	75	100	91.66	Spared	100	100	93.75	Spared	n/a	Spont. Other: spared Self Inhibit.: spared
5	38/M	stroke	100	100	100	Spared	100	100	100	Spared	n/a	Spont. Other: spared Self Inhibit.: spared
6	46/M	brain tumor	100	100	100	Spared	100	100	100	Spared	n/a	Spont. Other: spared Self Inhibit.: spared
7	59/M	stroke	87.5	100	100	Spared	100	88.88	93.75	Spared	n/a	Spont. Other: spared Self Inhibit.: spared
8	43/M	encephalitis meningitis	75	100	100	Spared	100	88.88	93.75	Spared	n/a	Spont. Other: spared Self Inhibit.: spared
9^b^	58/M	DAT	100	100	100	Spared	100	100	100	Spared	n/a	Spont. Other: spared Self Inhibit.: spared
10^a^	43/F	stroke	0	100	100	**Impaired**	87.5	100	100	Spared	Classical dissociation	Spont. Other: impaired Self Inhibit.: spared
11^b^	67/F	DAT	25	75	100	**Impaired**	100	100	100	Spared	Classical dissociation	Spont. Other: impaired Self Inhibit.: spared
12	74/F	stroke	75	100	100	Spared	0	100	100	**Impaired**	Classical dissociation	Spont. Other: spared Self Inhibit.: impaired
13	74/F	stroke	75	100	91.66	Spared	75	100	93.75	**Impaired**	No reliable dissociation	Spont. Other: spared Self Inhibit.: mildly impaired
14	54/F	stroke	100	100	100	Spared	75	100	100	**Impaired**	No reliable dissociation	Spont. Other: spared Self Inhibit.: mildly impaired
15 ^b^	59/F	DAT	37.5	50	91.66	**Impaired**	37.5	44.44	68.75	**Impaired**	No reliable dissociation	Either: Spont. Other: impaired Self Inhibit.: impaired or: other ToM impairment
16 ^b^	59/F	DAT	12.5	100	91.66	**Impaired**	12.5	66.66	81.25	**Impaired**	No reliable dissociation	Either: Spont. Other: impaired Self Inhibit.: impaired or: other ToM impairment
17 ^b^	73/F	DAT	25	50	91.66	**Impaired**	12.5	88.88	93.75	**Impaired**	No reliable dissociation	Either: Spont. Other: impaired Self Inhibit.: impaired or: other ToM impairment
18^a^	61/F	cerebral anoxia	0	100	91.66	**Impaired**	100	77.77	87.5	False negative	n/a	Spont. Other: to follow-up Self Inhibit.: to follow-up
19	57/F	stroke	50	50	91.66	Input info. deficit	100	100	100	Spared	n/a	Spont. Other: to follow-up Self Inhibit.: spared
20	40/M	stroke	100	50	91.66	False negative	87.5	100	100	Spared	n/a	Spont. Other: to follow-up Self Inhibit.: spared
21 ^b^	73/F	DAT	50	0	91.66	False negative	100	100	100	Spared	n/a	Spont. Other: to follow-up Self Inhibit.: spared

W = woman, M = man, DAT = degenerative dementia of Alzheimer’s type, % corr = % of correct responses, FB = false belief, TB = true belief, TB/MC = true belief/memory control, control trials include MC + filler for the Reality-unknown task or TB/MC + filler trials for the Reality-known task, n/a = not-applicable

^a^ patients who also participated in the study by Biervoye et al. [20]

^b^ patients who are also participated in another study (Biervoye et al., in preparation).

## Discussion

The two belief reasoning tasks used in this study were previously used in the literature to demonstrate the separability of two belief reasoning sub-processes: self-perspective inhibition and spontaneous other-perspective inference [17, 54–55]. The aim of this study was to adapt these two tasks for clinical purposes by collecting normative data and standardizing a diagnostic procedure. The results highlighted three important findings. First, the results obtained from the normative data collection showed a decrease in performance in one of the tasks as a function of age, namely the task that loaded most on spontaneous other-perspective inference demands. Secondly, for healthy participants, gender and socio-educational level had little to no impact in the two tasks. Thirdly, the use of the proposed standardized diagnostic procedure with brain damaged patients showed considerable heterogeneity in the integrity of the two targeted ToM processes following acquired brain damage. These results are discussed below.

### The effect of normal ageing and other demographic variables on false belief reasoning

Most previous studies which investigated the effects of age on ToM performance (including belief reasoning) came to the conclusion that performance decreases as age increases (e.g., [57, 69–70]. However, the results are not so clear-cut since some studies found no effect of age [71–72] or even a better performance as age increases [73]. These conflicting results have been explained in terms of methodological differences (tasks used, variability of the individuals’ characteristics, etc.). Nevertheless, recent meta-analyses support the notion of an overall decrease in ToM abilities with age (e.g., [34–74]) and our data is consistent with this general finding.

One of the main questions debated is whether such age effect on ToM performance is linked to a deterioration of general cognitive processes or to a deterioration of processes specific to ToM. On the one hand, some authors have shown that the decrease of performance in ToM tasks as a function of age is mainly observed in complex ToM tasks (such as for example, second-order false belief reasoning tasks) but not in simple ToM tasks (such as first-order false belief reasoning tasks) [69]. It has been thus proposed that older individuals do not have specific impairments in ToM but rather difficulties with the general cognitive resources required to solve complex ToM tasks [69–75]. Furthermore, some authors have shown that the decrease of ToM performance with age was mediated by alterations to general cognitive processes such as executive functions and speed of processing (e.g., [76–79]), updating information in working memory [70–72], logical reasoning and vocabulary level [76]), further supporting the idea that the ToM problems observed in older adults is in fact secondary to a general cognitive decline.

On the other hand, other authors have compared older adults’ peformance on ToM trials/tasks with closely matched control trials/tasks with as sole difference that control trials/tasks did not require mentalizing and found a larger age effect on the ToM trials/tasks [57, 70, 78] (for a recent meta-analysis, see also [34]), suggesting that the deterioration of ToM performance with age is due to genuine ToM difficulties rather than being secondary to a more general cognitive decline.

In our study, we found an effect of age on the false belief trials of the reality-unknown belief reasoning task but not on the false belief trials of the reality-known task. Irrespective of age, the false belief trials of the reality unknown-task were harder, indicating that the effect of age was observed in the most challenging belief reasoning condition. However, the effect of age was not significant on any of the control trials. This indicates that the older adults in our study were sensitive to the complexity of ToM reasoning rather than just sensitive to the complexity of the general task demands.

Few studies have investigated whether age affects different types of ToM process in the same way, especially as far as belief reasoning processes are concerned. One study used the same tasks as the ones we used in our study (but the long instead of the short version; [57]) and found that the older participants had lower performances than the younger participants only for the false belief trials in the reality-known task. The authors concluded that age affects selectively the ability to inhibit one’s own perspective. Our results do not fit with this profile of performance and interpretation as we found the opposite: an effect of age on the false trials of the reality-unknown task only, suggestive of a selective age effect on the ability to spontaneously track other people’s beliefs. These conflicting results could be explained by two methodological factors. Firstly, in both studies the analyses were performed at the group level. It is possible that the performance at the group level mask a high inter-individual variability in terms of which process is deteriorating. Different samplings of volunteers may thus lead to different patterns at the group level depending on the diversity of profiles in the sample. Secondly, these authors [57] have administrated the two tasks in the reverse order to our fixed order. In our study, the reality-unknown task was always presented before the reality-known task. In contrast, in the study by Bailey and Henry [57], the tasks were presented in two sessions and each session started with a block of trials of the reality-known task before presenting a block of the reality-unknown task. Given that the reality-known trials are presented before reality-unknown trials, once participants are presented with reality-unknown trials, they are likely to be primed by the « *reality-known* » task instruction (which is the task with the most explicit mentalizing instructions) to pay attention to the woman’s belief. Thus, the demands in spontaneous other-perspective inference could have been reduced in the study by Bailey and Henry [57]. Future studies will need to disentangle these as well as other potential reasons for the discrepant results.

Our analyses showed no significant effect of gender and socio-educational level on the two belief reasoning tasks. No effect of these variables was also observed on a verbal belief reasoning task in another study [28]. In the literature, the effect of gender in social cognition has mainly been investigated in relation to empathy and the results are not always consistent [35, 38]. Eisenberg and Lennon [38] explained that the effect of gender was mainly reported with self-reported measures (questionnaires) but not with direct measures (behavioral tasks), such as the ones employed here.

### Implication for models of cognitive theory of mind and clinical practice

Our results with a sample of brain damaged patients demonstrated a diversity of profiles in belief reasoning: some patients presenting with an impairment in self-perspective inhibition but not spontaneous other-perspective inference; others showed the reverse pattern; and in some cases, both processes were likely to be impaired. These results support the idea that ToM is not a unitary function [11,13] and more specifically that self-perspective inhibition and the spontaneous inference of other-perspective are sustained by distinct cognitive processes that can be selectively affected by brain damage to the right lateral prefrontal cortex in case of a selective deficit in self-perspective inhibition [17–19] and damage to the left posterior temporo-parietal junction in the case of a selective deficit in spontaneous other-perspective tracking [19–20,55].The separability of these two cognitive processes underlying ToM is also consistent with neuroimaging studies in healthy participants which have shown that self-perspective inhibition is sustained by the right lateral prefrontal cortex (e.g., [50–52]). Neuroimaging studies in healthy participants have yet to confirm the implication of the left temporo-parietal junction in spontaneous other-perspective tracking.

The observation of the heterogeneity in the patients’ performance also confirms the need to envisage rehabilitation programs that are tailored to the patients’ specific profiles. Little is known so far on the rehabilitation “ingredients” that make a program efficient. The diversity of profiles observed should prompt researchers to investigate the most effective exercises to restore or compensate for the impaired processes and it is highly likely that such exercises will differ in nature depending on the type of impairment. In some cases, the profile of the patient indicates that he or she has difficulties in processing the input information to belief reasoning. Rather than seeing this as an artefact of our tasks, we suggest that this could translate into difficulties processing the input to ToM reasoning in everyday life since most social interactions require integration of various pieces of information. For those patients, focusing first the rehabilitation on the general cognitive processes impaired rather than on belief reasoning could be more appropriate. Once the general cognitive resources have improved, the re-assessing of the belief reasoning abilities could then indicate whether impairments specific to belief reasoning exist and need to be taken care of.

It would be interesting to further examine the extent to which the impairment in self-perspective inhibition or spontaneous other-perspective tracking affects the reasoning about other mental states than beliefs. So far, the results suggest that self-perspective inhibition deficits reported in the type of belief reasoning tasks reported here extend to desire, emotion and visual perspective reasoning [19–20]. It is also important to note that the usefulness of the two belief reasoning tasks presented here is not limited to a population of patients with acquired brain damage but extends to other clinical populations with psychiatry (such as schizophrenia for example), developmental disorders (such as autism for example) or even populations of healthy children and adolescents. In particular, the analysis of the individual rather than the group profile may provide a much richer understanding of how ToM can be affected in those disorders. For example, using the same tasks and a similar diagnostic approach as the one described here, Maurage et collaborators [59] showed that while at the group level individuals with alcohol dependence show impaired ToM performance in both belief reasoning tasks, when looking at the individual profiles, only half of the sample showed a belief reasoning impairment. Furthermore, alcohol dependent individuals could either show an impairment in self-perspective inhibition or an impairment in tracking the other-perspective spontaneously, highlighting the potential limits of global and undifferentiated rehabilitation programs for these types of patients.

Finally, it is important to remind potential users of these tasks that clinical observations play an important role in refining the diagnosis based on the general three-step diagnostic approach.

### Limitations

Although the tasks presented here are very promising for providing a more fine-grained diagnosis of belief reasoning deficits, the study has some limitations. A first limitation concerns the impossibility to test some psychometric criteria of the tasks, such as their reliability and validity. The reliability of a task is measured by the stability of the results over time (“test-retest reliability”). Like with some tests assessing executive functions, this procedure is not feasible for our belief reasoning tasks. Indeed, administrating our tasks twice would strongly reduce the novelty feature inherent to the « reality-unknown » task and thereby reduce the demands in spontaneous other-belief tracking on the second presentation of the task. Assessing the convergent validity of our tasks involves comparing the performances of participants on these belief reasoning tasks with their performances obtained in another well-established test evaluating the same process. However, other ToM tasks used in the literature do not tax self-perspective inhibition and the spontaneous inference of others’ belief in the same way as our tasks.

A second limitation relates to the age limit of the participants (74 years). Future work should extend the normative data collection to older participants. Such extension would require most probably a larger number of healthy participants (given the possible greater heterogeneity of age effects) and stricter inclusion / exclusion criteria (to take into account the greater likelihood of neurodegenerative diseases as age increases).

## Conclusions

In sum, the present study describes the rationale and the normative data collection of two nonverbal tasks to assess the integrity and disentangle the cognitive processes involved in belief reasoning (i.e., self-perspective inhibition vs. spontaneous other-perspective inference). Data collected in healthy participants showed a deleterious effect of age in the performances of one of the tasks indicative of a possible larger effect of age on the ability to spontaneously track other people’s beliefs. The novel diagnostic procedure was applied to 21 brain-damaged patients and showed a diversity of profiles, including selective deficits. This latter finding shows the importance of taking into account the multiple facets of ToM during the diagnosis and rehabilitation of patients with suspected ToM deficits.

## References

[1] Premack D, Woodruff G. Does the chimpanzee have a theory of mind? Behavioral and Brain Sciences. 1978;4:515–26.

[2] Baron-Cohen S, Leslie AM, Frith U. Does the autistic child have a “theory of mind”? Cognition. 1985;21(1):37–46. doi: 10.1016/0010-0277(85)90022-8 2934210 10.1016/0010-0277(85)90022-8

[3] Senju A, Southgate V, White S, Frith U. Mindblind eyes: an absence of spontaneous theory of mind in Asperger syndrome. Science. 2009;325(5942):883–5. doi: 10.1126/science.1176170 19608858 10.1126/science.1176170

[4] Sprong M, Schothorst P, Vos E, Hox J, Van Engeland H. Theory of mind in schizophrenia meta-analysis. Br J Psychiatry. 2007;191(1):5–13. 17602119 10.1192/bjp.bp.107.035899

[5] Bosco FM, Capozzi F, Colle L, Marostica P, Tirassa M. Theory of Mind deficit in subjects with alcohol use disorder: An analysis of mindreading processes. Alcohol Alcohol. 2014;49(3):299–307. doi: 10.1093/alcalc/agt148 24064369 10.1093/alcalc/agt148

[6] Martín-Rodríguez JF, León-Carrión J. Theory of mind deficits in patients with acquired brain injury: a quantitative review. Neuropsychologia. 2010;48(5):1181–1191. doi: 10.1016/j.neuropsychologia.2010.02.009 20153762 10.1016/j.neuropsychologia.2010.02.009

[7] Poletti M, Enrici I, Adenzato M. Cognitive and affective Theory of Mind in neurodegenerative diseases: neuropsychological, neuroanatomical and neurochemical levels. Neurosci Biobehav Rev. 2012;36(9):2147–2164. doi: 10.1016/j.neubiorev.2012.07.004 22819986 10.1016/j.neubiorev.2012.07.004

[8] Kennedy DP, Adolphs R. The social brain in psychiatric and neurological disorders. Trends Cogn Sci. 2012;16(11):559–572. doi: 10.1016/j.tics.2012.09.006 23047070 10.1016/j.tics.2012.09.006 PMC3606817

[9] Henry JD, von Hippel W, Molenberghs P, Lee T, Sachdev PS. (2016). Clinical assessment of social cognitive function in neurological disorders. Nat Rev Neurol. 2016;12(1):28–39. doi: 10.1038/nrneurol.2015.229 26670297 10.1038/nrneurol.2015.229

[10] American Psychiatric Association Diagnostic and Statistical Manual of Mental Disorders Fifth Edition American Psychiatric Publishing; 2013.

[11] Schurz M, Radua J, Aichhorn M, Richlan F, Perner J. Fractionating theory of mind: A meta-analysis of functional brain imaging studies. Neurosci Biobehav Rev. 2014;42:9–34. doi: 10.1016/j.neubiorev.2014.01.009 24486722 10.1016/j.neubiorev.2014.01.009

[12] Van Overwalle F. Social cognition and the brain: a meta-analysis.Human brain mapping. 2009;30(3):829–858. doi: 10.1002/hbm.20547 18381770 10.1002/hbm.20547 PMC6870808

[13] Samson D. Reading other people's mind: Insights from neuropsychology. J Neuropsychol 2009;3(1):3–16. doi: 10.1348/174866408X377883 19338713 10.1348/174866408X377883

[14] Shamay-Tsoory SG, Aharon-Peretz J. Dissociable prefrontal networks for cognitive and affective theory of mind: a lesion study. Neuropsychologia. 2007;45(13):3054–3067. doi: 10.1016/j.neuropsychologia.2007.05.021 17640690 10.1016/j.neuropsychologia.2007.05.021

[15] Mitchell RL, Phillips LH. The overlapping relationship between emotion perception and theory of mind. Neuropsychologia. 2015;70:1–10. doi: 10.1016/j.neuropsychologia.2015.02.018 25687032 10.1016/j.neuropsychologia.2015.02.018

[16] Sabbagh MA, Moulson MC, Harkness KL. Neural correlates of mental state decoding in human adults: An event-related potential study. J Cogn Neurosci. 2004;16(3):415–426. doi: 10.1162/089892904322926755 15072677 10.1162/089892904322926755

[17] Samson D, Apperly IA, Kathirgamanathan U, Humphreys GW. Seeing it my way: a case of a selective deficit in inhibiting self-perspective. Brain. 2005;128(5): 1102–1111. doi: 10.1093/brain/awh464 15774506 10.1093/brain/awh464

[18] Samson D, Houthuys S, Humphreys GW. Self-perspective inhibition deficits cannot be explained by general executive function difficulties. Cortex. 2015;70: 189–201. doi: 10.1016/j.cortex.2014.12.021 25752979 10.1016/j.cortex.2014.12.021

[19] Samson D, Apperly IA, Humphreys GW. Error analyses reveal contrasting deficits in “theory of mind”: Neuropsychological evidence from a 3-option false belief task. Neuropsychologia. 2007;45(11):2561–2569. doi: 10.1016/j.neuropsychologia.2007.03.013 17451756 10.1016/j.neuropsychologia.2007.03.013

[20] Biervoye A, Dricot L, Ivanoiu A, Samson D. Impaired spontaneous belief inference following acquired damage to the left posterior temporo-parietal junction. Soc Cogn Affect Neurosci. 2016;nsw076 doi: 10.1093/scan/nsw076 27317925 10.1093/scan/nsw076 PMC5040917

[21] Bosco F. M., Gabbatore I., Tirassa M., & Testa S. (2016). Psychometric properties of the Theory of Mind Assessment Scale in a sample of adolescents and adults. Frontiers in psychology, 7. 27242563 10.3389/fpsyg.2016.00566 PMC4860419

[22] Bosco F. M., Colle L., De Fazio S., Bono A., Ruberti S., & Tirassa M. (2009). Thomas: An exploratory assessment of Theory of Mind in schizophrenic subjects. Consciousness and cognition, 18(1), 306–319. doi: 10.1016/j.concog.2008.06.006 18667334 10.1016/j.concog.2008.06.006

[23] Ekman P, Friesen WV. Pictures of Facial Affect. Consulting Psychologists Press;1976.

[24] Young A, Perrett D, Calder A, Sprengelmeyer R, Ekman P. Facial Expressions of Emotion- Stimuli and Tests (FEEST) Thames Valley Test Company;2002.

[25] Baron-Cohen S, Wheelwright S, Hill J, Raste Y, Plumb I. The “Reading the Mind in the Eyes” Test revised version: a study with normal adults, and adults with Asperger syndrome or high-functioning autism. J Child Psychol Psychiatry. 2001;42(2):241–51. doi: 10.1111/1469-7610.00715 11280420

[26] Sarfati Y, Brunet E, Hardy-Baylé MC. Comic-strip Task: Attribution of Intentions to Others. Service de Psychiatrie Adulte, Hôpital de Versailles, Le Chesnay, France; 2003.

[27] Chiavarino C, Apperly IA, Humphreys GW. Distinguishing intentions from desires: Contributions of the frontal and parietal lobes. Cognition. 2010;117(2): 203–216. doi: 10.1016/j.cognition.2010.08.012 20817149 10.1016/j.cognition.2010.08.012

[28] Desgranges B, Laisney M, Bon L, Duval C, Mondou A, Bejanin A., et al (2012). TOM-15: Une épreuve de fausses croyances pour évaluer la théorie de l'esprit cognitive. Revue de neuropsychologie. 2012;4(3):216–220. doi: 10.1684/nrp.2012.0232$

[29] Stone VE, Baron-Cohen S, Knight RT. Frontal lobe contributions to theory of mind. Journal of cognitive neuroscience. 1998;10(5):640–656. 9802997 10.1162/089892998562942

[30] Happé FG. An advanced test of theory of mind: Understanding of story characters' thoughts and feelings by able autistic, mentally handicapped, and normal children and adults. J Autism Dev Disord. 1994;24(2):129–154. 8040158 10.1007/BF02172093

[31] White S, Hill E, Happé F, Frith U. Revisiting the strange stories: Revealing mentalizing impairments in autism. Child Dev. 2009;80(4):1097–1117. doi: 10.1111/j.1467-8624.2009.01319.x 19630896 10.1111/j.1467-8624.2009.01319.x

[32] Kanai R, Rees G. The structural basis of inter-individual differences in human behaviour and cognition. Nat Rev Neurol. 2011;12(4):231–242. doi: 10.1038/nrn3000 21407245 10.1038/nrn3000

[33] Reniers R, Corcoran R, Drake R, Shryane NM, Völlm BA. The QCAE: a Questionnaire of Cognitive and Affective Empathy. J Pers Assess. 2011;93(1): 84–95. doi: 10.1080/00223891.2010.528484 21184334 10.1080/00223891.2010.528484

[34] Henry JD, Phillips LH, Ruffman T, Bailey PE. A meta-analytic review of age differences in theory of mind. Psychol Aging. 2013;28(3):826 doi: 10.1037/a0030677 23276217 10.1037/a0030677

[35] Baron-Cohen S, Wheelwright S. The empathy quotient: an investigation of adults with Asperger syndrome or high functioning autism, and normal sex differences. J Autism Dev Disord. 2004;34(2):163–75. 15162935 10.1023/b:jadd.0000022607.19833.00

[36] Krach S, Blümel I, Marjoram D, Lataster T, Krabbendam L, Weber J, et al Are women better mindreaders? Sex differences in neural correlates of mentalizing detected with functional MRI. BMC Neurosci. 2009;10(1):1. doi: 10.1186/1471-2202-10-9 19193204 10.1186/1471-2202-10-9 PMC2667181

[37] Veroude K, Jolles J, Croiset G, Krabbendam L. Sex differences in the neural bases of social appraisals. Soc Cogn Affect Neurosci. 2014;9(4):513–519. doi: 10.1093/scan/nst015 23386740 10.1093/scan/nst015 PMC3989134

[38] Eisenberg N, Lennon R. Sex differences in empathy and related capacities. Psychol Bull. 1983;94(1):100 doi: 10.1037/0033-2909.94.1.100

[39] Wimmer H, Perner J. Beliefs about beliefs: Representation and constraircing function of wrong beliefs in young children’s understanding of deception. Cognition. 1983;13:103–128. doi: 10.1016/0010-0277(83)90004-5 6681741 10.1016/0010-0277(83)90004-5

[40] Bloom P, German TP. Two reasons to abandon the false belief task as a test of theory of mind. Cognition. 2000;77(1): B25–B31. doi: 10.1016/S0010-0277(00)00096-2 10980256 10.1016/s0010-0277(00)00096-2

[41] Wellman HM, Liu D. Scaling of theory-of-mind tasks. Child Dev. 2004;75(2):523–541. doi: 10.1111/j.1467-8624.2004.00691.x 15056204 10.1111/j.1467-8624.2004.00691.x

[42] Apperly IA, Samson D, Humphreys GW. (2009). Studies of adults can inform accounts of theory of mind development. Dev Psychol. 2009;45(1):190–201. doi: 10.1037/a0014098 19210001 10.1037/a0014098

[43] Onishi KH, Baillargeon R. Do 15-month-old infants understand false beliefs? Science. 2005;308;255–258. doi: 10.1126/science.1107621 15821091 10.1126/science.1107621 PMC3357322

[44] Schneider D, Nott ZE, Dux PE. Task instructions and implicit theory of mind. Cognition. 2014;133(1):43–47. doi: 10.1016/j.cognition.2014.05.016 24955887 10.1016/j.cognition.2014.05.016

[45] Birch SA, Bloom P. The curse of knowledge in reasoning about false beliefs. Psychol Sci. 2007;18(5):382–386. doi: 10.1111/j.1467-9280.2007.01909.x 17576275 10.1111/j.1467-9280.2007.01909.x

[46] Leslie A. M., & Polizzi P. (1998). Inhibitory processing in the false belief task: Two conjectures. Developmental Science, 1(2), 247–253.

[47] Leslie A. M., Friedman O., & German T. P. (2004). Core mechanisms in ‘theory of mind’. Trends in cognitive sciences, 8(12), 528–533. doi: 10.1016/j.tics.2004.10.001 15556021 10.1016/j.tics.2004.10.001

[48] Aboulafia-Brakha T, Christe B, Martory MD, Annoni JM. Theory of mind tasks and executive functions: a systematic review of group studies in neurology. J Neuropsycho. 2011;5(1):39–55. doi: 10.1348/174866410X533660 21366886 10.1348/174866410X533660

[49] Abraham A, Rakoczy H, Werning M, von Cramon DY, Schubotz RI. Matching mind to world and vice versa: functional dissociations between belief and desire mental state processing. Soc Neurosci. 2010;5(1):1e18 doi: 10.1080/17470910903166853 19670085 10.1080/17470910903166853

[50] Hartwright CE, Apperly IA, & Hansen PC. Multiple roles for executive control in belief-desire reasoning: distinct neural networks are recruited for self perspective inhibition and complexity of reasoning. Neuroimage. 2012;61(4):921–930. doi: 10.1016/j.neuroimage.2012.03.012 22440654 10.1016/j.neuroimage.2012.03.012

[51] Hartwright CE, Apperly I.A, & Hansen PC. The special case of self-perspective inhibition in mental, but not non-mental, representation. Neuropsychologia. 2015;67: 183–192. doi: 10.1016/j.neuropsychologia.2014.12.015 25527113 10.1016/j.neuropsychologia.2014.12.015

[52] Van der Meer L, Groenewold NA, Nolen WA, Pijnenborg M, Aleman A. Inhibit yourself and understand the other: neural basis of distinct processes underlying Theory of Mind. Neuroimage. 2011;56(4):2364–2374. doi: 10.1016/j.neuroimage.2011.03.053 21440642 10.1016/j.neuroimage.2011.03.053

[53] Vogeley K, Bussfeld P, Newen A, Herrmann S, Happe F, Falkai P, et al Mind reading: neural mechanisms of theory of mind and self-perspective. Neuroimage. 2001;14:170–181. doi: 10.1006/nimg.2001.0789 11525326 10.1006/nimg.2001.0789

[54] Apperly IA, Samson D, Chiavarino C, Humphreys GW. Frontal and temporo-parietal lobe contributions to theory of mind: neuropsychological evidence from a false-belief task with reduced language and executive demands. J Cogn Neurosci. 2004;16(10):1773–1784. doi: 10.1162/0898929042947928 15701227 10.1162/0898929042947928

[55] Samson D, Apperly IA, Chiavarino C, Humphreys GW. Left temporoparietal junction is necessary for representing someone else’s belief. Nat Neurosci. 2004;7(5):499–500. doi: 10.1038/nn1223 15077111 10.1038/nn1223

[56] Le Bouc R, Lenfant P, Delbeuck X, Ravasi L, Lebert F, Semah F et al My belief or yours? Differential theory of mind deficits in frontotemporal dementia and Alzheimer’s disease. Brain. 2012;135(10):3026–3038. 23065791 10.1093/brain/aws237

[57] Bailey PE, Henry JD. (2008). Growing less empathic with age: Disinhibition of the self-perspective. J Gerontol B Psychol Sci Soc Sci. 2008;63(4):219–226. doi: 10.1093/geronb/63.4.P219 10.1093/geronb/63.4.p219 18689763

[58] Schneider D, Slaughter VP, Bayliss AP, Dux PE. A temporally sustained implicit theory of mind deficit in autism spectrum disorders. Cognition. 2013;129(2):410–417. doi: 10.1016/j.cognition.2013.08.004 23994318 10.1016/j.cognition.2013.08.004

[59] Maurage F, Timary P, Tecco JM., Lechantre S, Samson D. Theory of mind difficulties in patients with alcohol dependence: beyond the prefrontal cortex dysfunction hypothesis. Alcohol Clin Exp Res. 2015;39(6):980–988. doi: 10.1111/acer.12717 26033203 10.1111/acer.12717

[60] Folstein MF, Folstein SE, McHugh PR. “Mini-mental state”. A practical method for grading the cognitive state of patients for the clinician. J Psychiatr Res.1975;12(3):189–198. 1202204 10.1016/0022-3956(75)90026-6

[61] Morris JC, Mohs RC, Rogers H, Fillenbaum G, Heyman A. Consortium to establish a registry for Alzheimer's disease (CERAD) clinical and neuropsychological assessment of Alzheimer's disease. Psychopharmacol Bull. 1988;24(4):641–52. 3249766

[62] Reitan RM. The relation of the trail making test to organic brain damage. J Consult Psychol. 1955;19(5):393–394 13263471 10.1037/h0044509

[63] Cardebat D, Doyon B, Puel M, Goulet P, Joanette Y. Formal and semantic lexical evocation in normal subjects. Performance and dynamics of production as a function of sex, age and educational level. Acta Neurol Belg.1990;90(4):207–217. 2124031

[64] de Partz MP, Bilocq V, De Wilde V, Seron X, Pillon A. LEXIS: Tests pour l’évaluation des troubles lexicaux chez la personne aphasique Marseille: Solal 2001.

[65] Call J, Tomasello M. A nonverbal false belief task: The performance of children and great apes. Child Dev. 1999,70(2):381–395. doi: 10.1111/1467-8624.00028 10218261 10.1111/1467-8624.00028

[66] Crawford JR, Garthwaite PH, Gray CD. Wanted: Fully operational definitions of dissociations in single-case studies. Cortex. 2003;39(2):357–370. doi: 10.1016/S0010-9452(08)70117-5 12784893 10.1016/s0010-9452(08)70117-5

[67] Field A. Discovering Statistics using SPSS (sd edition). Sage Publications: London 2015.

[68] Cousineau D. Confidence intervals in within-subject designs: A simpler solution to Loftus and Masson’s method. Tutor Quant Methods Psychol. 2005; 1(1):42–45.

[69] McKinnon MC, Moscovitch M. Domain-general contributions to social reasoning: Theory of mind and deontic reasoning re-explored. Cognition. 2007;102(2):179–218. doi: 10.1016/j.cognition.2005.12.011 16480974 10.1016/j.cognition.2005.12.011

[70] Phillips LH, Bull R, Allen R, Insch P, Burr K, Ogg W. (2011). Lifespan aging and belief reasoning: Influences of executive function and social cue decoding. Cognition, 120, 236–247. doi: 10.1016/j.cognition.2011.05.003 21624567 10.1016/j.cognition.2011.05.003

[71] MacPherson SE, Phillips LH, Della Sala S. Age, executive function and social decision making: a dorsolateral prefrontal theory of cognitive aging. Psychol Aging. 2002;17(4):598 doi: 10.1037/0882-7974.17.4.598 12507357

[72] Saltzman J, Strauss E, Hunter M, Archibald S. Theory of mind and executive functions in normal human aging and Parkinson's disease. J Int Neuropsychol Soc. 2000;6(07):781–788. 11105468 10.1017/s1355617700677056

[73] Happé FG, Winner E, Brownell H. The getting of wisdom: Theory of mind in old age. Dev Psychol. 1998;34:358–362. 9541787 10.1037//0012-1649.34.2.358

[74] Sandoz M, Démonet JF, Fossard M. Theory of mind and cognitive processes in aging and Alzheimer type dementia: a systematic review. Aging Ment Health. 2014;18(7):815–827. doi: 10.1080/13607863.2014.899974 24697253 10.1080/13607863.2014.899974

[75] Slessor G, Phillips LH, Bull R. Exploring the specificity of age-related differences in theory of mind tasks. Psychology and aging. 2007;22(3):639 doi: 10.1037/0882-7974.22.3.639 17874961 10.1037/0882-7974.22.3.639

[76] Charlton RA, Barrick TR, Markus HS, Morris RG. Theory of mind associations with other cognitive functions and brain imaging in normal aging. Psychol Aging. 2009;24:338–348. doi: 10.1037/a0015225 19485652 10.1037/a0015225

[77] Duval C, Piolino P, Bejanin A, Eustache F, Desgranges B. Age effects on different components of theory of mind. Conscious Cogn. 2011;20(3):627–642. doi: 10.1016/j.concog.2010.10.025 21111637 10.1016/j.concog.2010.10.025

[78] German TP, Hehman JA. Representational and executive selection resources in ‘theory of mind’: Evidence from compromised belief-desire reasoning in old age. Cognition. 2006;101:129–152. doi: 10.1016/j.cognition.2005.05.007 16288734 10.1016/j.cognition.2005.05.007

[79] Li X, Wang K, Wang F, Tao Q, Xie Y, Cheng Q. Aging of theory of mind: The influence of educational level and cognitive processing. International Journal of Psychology. 2013;48(4):715–727. doi: 10.1080/00207594.2012.673724 22515730 10.1080/00207594.2012.673724

